# CGF Treatment of Leg Ulcers: a Randomized Controlled Trial

**DOI:** 10.1515/med-2019-0113

**Published:** 2019-12-24

**Authors:** Bruno Amato, Michele Angelo Farina, Silvana Campisi, Marino Ciliberti, Vincenzo Di Donna, Anna Florio, Antonino Grasso, Rosario Miranda, Francesco Pompeo, Eleonora Farina, Raffaele Serra, Roberto Cirocchi, Francesca Calemma, Aldo Rocca, Rita Compagna

**Affiliations:** 1 Department of Clinical Medicine and Surgery, University Federico II of Naples, Italy, via S. Pansini, 5, 80131 Naples, Italy; 2 Department of Clinical Medicine and Surgery, University Federico II of Naples, Naples, Italy; 3 Interuniversity Center of Phlebolymphology (CIFL), International Research and Educational Program in Clinical and Experimental Biotechnology, Catanzaro, Italy; 4 Vascular Surgery Unit, Villa Del Sole Hospital 81100 Caserta, Italy; 5 Transfusion Immunohematology Service, S.Andrea Hospital, 00192 Rome, Italy; 6 Ulcer Service Program, Castellammare Di Stabia, Naples, Italy; 7 Medical Center Regeneration Home, Corato, Foggia,Italy; 8 Department of Cardio-thoracic and Respiratory Sciences, University of Campania “Luigi Vanvitelli”, Naples, Italy; 9 Vascular Surgery Unit, University of Catania, “Policlinico, Vittorio Emanuele” P.O. G. Rodolico , Catania, Italy; 10 Angiology Service ASL NA 3 Sud, Nola District, 80035 Nola, Napoli, Italy; 11 Vascular Surgery Unit, Neurologic Mediterranean Institute Neuromed, 86077 Pozzilli, Isernia, Italy; 12 Department of Medical and Surgical Science, University Magna Graecia of Catanzaro, Catanzaro, Italy; 13 Department of Surgical and Biomedical Sciences, Division of Week surgery, S. Maria Hospital, Terni, Italy

**Keywords:** Concentrated Growth Factors, CGF, Platlet Rich Plasma, PRP, Mixed ulcers, Vascular ulcers, Leg ulcers

## Abstract

**Background:**

Concentrated Growth Factors (CGF) is a concentration of second generation autologous growth factors compared to platelet rich plasma (PRP) and represents a multifactorial stimulation system that can be used for the management and treatment of chronic skin ulcers.

**Aim:**

The aim of this work is to evaluate the additional benefits of the CGF compared to the standard of dressing and its effects on the dynamics of the healing process.

**Methods:**

Autologous CGFs were obtained from 100 patients with chronic mixed ulcers (venous ulcers in patients with II stage claudication) of the lower limbs in a multicentric controlled randomized study.

**Results:**

The results showed a significant advantage in the use of CGF in association with cleansing and selective compression in the healing time and stabilization of mixed ulcers of the lower extremities.

**Conclusions:**

These results support the CGF’s clinical use for improving clinical outcomes in mixed ulcers of the legs.

## 1 Introduction

Concentrated Growth Factors (CGF) is a second-generation mixture of autologous growth factors compared to platelet rich plasma (PRP). It represents a multifactorial stimulation system as it uses all phases of the blood. It is proposed for the ancillary treatment of chronic skin ulcers.

The advantages of CGF found in literature are the immediate hemostatic action, biocompatibility, fast epithelial regeneration (by stimulating angiogenesis and promoting the synthesis of collagen protein, so it also promotes tissue healing) and a high antimicrobial effect due to the high concentration of leukocytes [1, 2, 3, 4].

Progress in the management of chronic skin wounds has led to high rates of recovery [5, 6, 7] but further works are required in order to improve the effectiveness of treatment protocols and the comfort and safety of patients also.

The CGF represents an evolution of the PRP. Its application is progressively spreading in the clinical field. CGF and PRP are based on the release of platelet growth factors, mainly contained in alpha granules, which exert a stimulating action on the reparative processes of skin lesions. Few studies have been published, hence the literature provides a low level of similarity in cases and protocols [8,9].

### 1.1 Aim of the study

The current work aims to evaluate the additional benefits of the CGF compared to the standard dressing and compression of chronic leg ulcers and its effects on the dynamics of the healing process.

## 2 Materials and Methods

A multi-centre randomized controlled trial has been performed for patients with mixed ulcers: i.e. on a population with venous insufficiency (whose diagnosis was evidenced by a clinical and an echo-doppler examination of venous district of the lower limbs, as well as by the typical ulcer location - the medial region of the ankle) and chronic obstructive arterial disease (whose diagnosis was also evidenced by a clinical and instrumental examination showing the second stage of Rutherford classification peripheral arterial disease, with an ankle / arm pressure index between 0.5 and 0.9 at the affected limb). The protocol consisted of the application of CGF in addition to a standard wound dressing and elastic compression, performed once a week, for a 12 weeks period (or less in case of ulcer healing) and followed by a 6 months follow-up. None of the patients enrolled underwent surgery for venous or arterial disease, as they were considered not suitable or they refused the treatment: these kinds of mixed ulcers are usually characterized by a significantly slower healing time compared with ulcers that are just venous. The trial was preliminarily approved by the Institutional Review Board Independent Ethics Committee (IRB-IEC) as the Ethics Committee of the Inter-University Centre of Phlebo-lymphology (CIFL), with a positive result (protocol: ER: ALL.2016.03A). The study was conducted by recruiting patients from among ten vascular centres of Southern Italy - reported in “Supporting Info”- under the control of the authors as members of the “SIMCRI Group for the study of CGF in the treatment of Mixed Ulcers”, and coordinated by the CIFL researcher’s group.

It should be noted that both comparative study groups have previously applied procedures in clinical use: specifically, we refer to CGF as a recent method of preparing platelet gel that enriches the dressing of growth factors present in platelet granules [10, 11, 12, 13, 14, 15].

It is also correct to specify that no significant methodological changes have been made after the start of the trial.

Patient enrollment took place between December 2016 and December 2017. The clinical trial and related evaluation analysis were completed in July 2018. The primary end-point was identified in the reduction of the surface of the ulcerative wounds within 12 weeks of treatment. Secondary end-points were the presence and variation of ulceration pain and the reduction of bacterial load during treatment.

### 2.1 Patients enrollment and inclusion criteria

Following a selection of 143 patients with mixed ulcers of the legs initially enrolled and after excluding 43 patients (of whom 36 did not reach the inclusion criteria for duration of ulcers, 5 declined to participate to the study protocol and 2 were excluded due to a severe degree of wound infection), 100 patients (64 women and 36 males, mean age 68 +/- 8 years) with chronic mixed ulcers of the legs (over three months lasting) were recruited into the Vascular Units participating in the study. These patients were randomized into two homogeneous groups: a group (A) of 53 patients who received CGF dressing in addition to standard treatment, and a group (B) of 47 patients which received a standard wound treatment as later described.

As a criterion for a correct management of the lesion, the principles of wound bed preparation have been applied. [16,17], and an accurate cleansing of the ulcer from necrotic material was carried out in a sterile way during each dressing.

All the patients participating in the study were informed about the aims of the study and an informed consent was provided and stored in the records of each participating centre. Once informed consent has been obtained, a clinical data collection form, common to all participating centres, has been compiled.

### 2.2 Preliminary exclusion criteria

The exclusion criteria applied in the recruitment of patients were the following: recent outcomes of myocardial infarction, dialysis, a serious infection of the lesion (with extension to the surrounding tissues), reduced life expectancy, cachexia. Drugs already in use for other pathologies of each patient were not considered exclusion criteria, except in cases of significant modification of the therapy, necessary during the study itself. The reasons for the exclusion from the study were reported.

**Figure 1:**
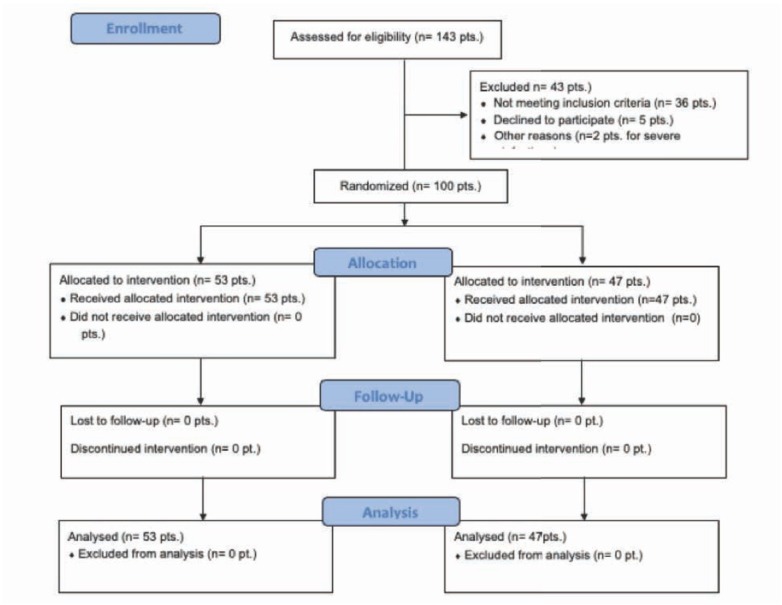
Consort checlist

**Table 1:** Clinical features of Group A and Group B patients with mixed limb ulcers.

Pts serie n.100	Group A n. 53	Group B n. 47
Male/Female	M = 18 /F =35	M = 16 / F =31
Mean Age	M = 62+/-9	M = 68+/-5
	F = 71+/-12	F = 69+/-10
Wound Size (cm^2^)	24+/-16	22+/-9

The exclusion criteria expected for the study was the following: extension or onset of severe ulcer infection and amputation or revascularization surgery.

### 2.3 Outcomes

The primary outcome of the study was surface reduction and ulcer healing after 12 weeks of treatment, while secondary outcomes were sought in the reduction of wound-related pain and in the reduction of the bacterial component in the ulcer: these outcomes did not change during the trial.

### 2.4 Ulcers examination

In order to define the characteristics of the lesion in terms of surface, the presence of fibrinous material (slough) or granulation tissue, comparable and quantifiable photographic images of the lesions were acquired, subjected to the evaluation of two external neutral observatories: at the beginning of the protocol and for each medication (both in the treatment and group phases in the monthly follow-up).

Complete ulcer healing was defined by both clinical and photographic documentation of complete skin epithelialization.

### 2.5 Preparation of the CGF

CGF was produced by the patient’s blood without addition of exogenous substances through the use of a special centrifuge Phase Separator (Medifuge MF 200® - Silfradent, Italy). After taking 10 ml of blood from an arm vein, under aseptic conditions, the blood centrifugation / separation process followed an automatic program with alternate speeds that prevented platelet degranulation. The centrifugation duration was 14 minutes, with the following program: - 2 minutes at 2700 rotations per minute (rpm) (to obtain the separation of blood components); - 4 minutes at 2400 rpm (to obtain cell lysis); - 4 minutes at 2700 rpm (to get the aggregation of the blood components); - 3 minutes at 3000 rpm (to get homogenization of blood components).

Other characteristics of centrifugation were:

1. Acceleration in about 29/30 sec. from 0 to 2700 rpm with Fg 25 (to avoid any damage to the blood components);
2. Stop in 33 sec. from 3000 to 0 rpm with Fg 87/88 (to avoid the mixing of the fractions obtained);
3. Use of glass test-tube (CE certificate 0476) (“red cap”) to obtain a product with characteristics of gel, for elastic and suturing membranes.4. Use of glass tube with red cap with addition of 18/20 units Sodium Heparin (“Sodium Heparin”) to keep all phases in liquid state: allows to obtain different fractions (serum, fibrin-rich plasma and platelet-poor, fibrinrich and platelet-rich plasma, leukocyte layers, mononuclear cells and fraction with erythrocytes) in the liquid state, therefore not gelled. This tube “Sodium Heparin” allows for the different fractions in liquid to form at room temperature for about 8 hours at a temperature of 4 ° C for about 40 hours. In addition, Sodium Heparin aggregates the platelets for a better release of the growth factors [18].

## 3 Randomization

Patients were randomized by selecting enrolled patients for Group A (CGF supplementary treatment) and Group B (standard treatment as control group), by a software-generated procedure provided by a computerized randomization program with audit trial. Randomization was implemented by the single coordinator for randomization and was controlled by the principal investigator at University of Naples “Federico II”. Randomization was also indicated in the patient record.

### 3.1 Topical ulcer therapy

#### 3.1.1 Standard dressing (group A)

The dressing was carried out with the following protocol: 1. Cleansing with physiological solution at a temperature between 34 and 37° C; 2. Dakin’s solution pack (sodium hypochlorite diluted 0.05%) for 5 min.; 3. Direct application of the CGF gel on the wound; 4. Covering of CGF with gauze containing hyaluronic acid; 5. Subsequent covering with an advanced absorbent and impermeable medication (Allevyn Adhesive ®); 6. Application of a containment sock (day and night) and of an elastic leg (18 mmHg at the ankle, personal measure), only during the day.

#### 3.1.2 Standard medication (group B)

The dressing was carried out with the same protocol, devoid of only point 3, the CGF application.

The dressings were scheduled every week for twelve weeks, and a follow- up of a further twelve weeks where a standard weekly dressing was applied to all patients, if the healing of the ulcers had not yet occurred. When healing was completed, elastic compression already in place (with monthly check- up) was prescribed.

### 3.2 Follow up

Dressings and follow-up have been performed at the participating centres, by those responsible for the protocol application. Failure to comply with the prescribed dressing time for an advance or delay of more than one day, or the accumulation of deviations from the scheduled dressing cadence above a total of two days throughout the duration of the protocol were considered a lack of adherence to the protocol and valid reasons for exclusion from the study.

### 3.3 Pain assessment

**Figure 2:**
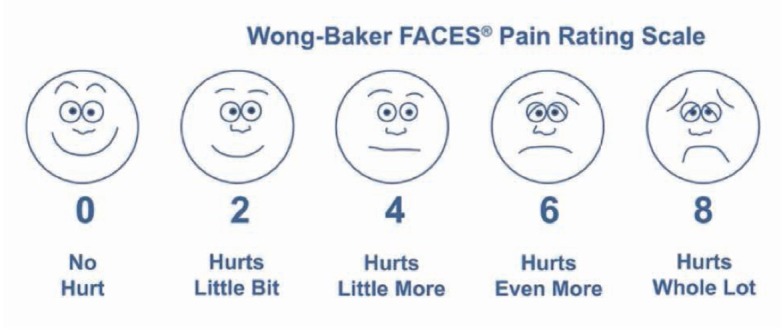
Wong-Baker scale for pain measurement.

Pain assessment was performed at enrolment and at each dressing change by evaluating pain intensity according to the Wong-Baker scale, as in Fig.2, using its numerical values, and recording on the personal data sheet of each patient its reported intensity, as well as the characteristics of location, duration, concomitance and associated symptoms.

Patients, however, were allowed to treat pain with home therapies using non-steroidal anti-inflammatory drugs, when necessary.

### 3.4 Evaluation of the bacterial load

The evaluation of the bacterial load was performed, with a semi-quantitative buffer, in all the patients enrolled in the study during the first dressing (T0) and during the following dressings after 3-6-9 weeks (T3-T6-T9). Bacterial growth values of 4+ or higher were considered positive for wound infection, while lower values were considered indicative of a non-infected wound.

### 3.5 Statistical analysis

All statistical analyses derived from this study were performed using Medical statistical software. Visual histograms and analytical methods (Student’s t-test) were used for the determination of normal distribution in all the expected outcomes. The statistical significance threshold was set to * P <0.05. Continuous variables were expressed as mean ± standard mean error. Paired values for planimetric data, including wound area (as cm^_2_^), were compared with paired samples t-test among consecutive times of the study period.

## 4 Results

The treatment of wound dressings, according to the procedures for group A and group B of patients, associated with elastic compression, was correctly performed for 12 weeks in all recruited patients. In group A, complete wound healing was found in all patients (53/53) during the first 12 weeks of observation: in 11 patients. (20.7%) after 5 weeks, in 14 patients (26.4%) in 6 weeks, in 10 patients (18.8%) in 7 weeks, in 8 patients (15.0%) in 8 weeks, in 7 patients (13.2%) in 9 weeks, in 2 patients (3.7%) in 10 weeks, and in 1 patient (1.8%) in 11 weeks. Ulcers healing in group B occurred in the first phase of the study (12 week interval from first observation) only in 32 out of this 47 patients (68% of cases), with the following timing: in 2 patients (4.2%) in the first 5 weeks; in 1 patient (2.1%) in 6 weeks, in 3 patients (6.3%) in 8 weeks, in 5 patients (10.5%) in 9 weeks, in 7 patients (14.8%) in 10 weeks, in 6 patients (13.9%) in 11 weeks, in 8 patients (18.6%) in 12 weeks, while in 15 patients (31.9%) wound healing was achieved over 12 weeks.

Evolution of the healing times in the two groups of treated patients is described in Fig. 2.Comparing the two groups in consecutive weeks, the surface of the ulcers did not show a significant reduction in comparative control after the first week (p = 0.014), while it was possible to evaluate significant reductions in all consecutive comparative controls occurred between the first and second week (p = 0.004), and then between subsequent weeks (p <0.001).

The overall evaluation of pain intensity by means of the Visual Analogue Scale (VAS), using the Wong-Baker scale, initially homogeneous in the two study groups (5.38 +/- 1.35 in Group A and 5.16 +/- 1.94 in group B) showed a significant improvement (p <0.001) of pain symptoms in Group A patients compared to those of Group B, starting from the second week of treatment (Group A = 2.88 +/- 0.83, Group B = 5.26+ /-1.14), progressively improved in the following weeks (Tab. III), with the complete disappearance of pain that accompanied the healing of the ulcer at an average 3.4 weeks.

**Table 2:** Median percentage of reduction of wound area for repeated comparisions during the observational period (W1_→_W2) in patients treated with CGF dressing + compression (Group A) and standard dressing + compression (Group B).

% of reduction	W1	W2	W3	W4	W5	W6	W7	W8	W9	W10	W11	W12
Group A	12	23	28	35	46	52	58	64	72	85	94	100
(n. of pts.)					(42/53)	(28/53)	(18/53)	(10/53)	(3/53)	(1/53)	(1/53)	
Group B	10	12	15	19	22	28	36	41	49	57	65	71
(n. of pts.)					(45/47)	(44/47)	(44/47)	(41/47)	(36/47)	(29/47)	(23/47)	(15/47)
p-value for comparisions		p=0.001	p=0.001	p=0.001	p=0.001	p=0.001	p=0.001	p=0.001	p=0.001			

**Table 3:** Median reduction of pain for repeated comparisions during the observational period (W1_à_W2) in patients treated with CGF dressing + compression (Group A) and standard dressing + compression (Group B).

% of pain reduction	W1	W2	W3	W4	W5	W6	W7	W8	W9	W10	W11	W12
Group A	5.38	2.12	1.82	1.14	0.76	0.54	0.47	0.40	0.46	-	-	-
(n. 53 pts.)					(42/53)	(28/53)	(18/53)	(10/53)	(3/53)	(1/53)	(1/53)	
Group B	5.16	4.78	4.55	3.81	3.22	2.65	2.52	2.41	1.96	1.88	1.57	1.45
(n. 47 pts.)					(45/47)	(44/47)	(44/47)	(41/47)	(36/47)	(29/47)	(23/47)	(15/47)
p-value for comparisions	p=0.014	p=0.004	P<0.001	P<0.001	P<0.001	P<0.001	P<0.001	P<0.001	P<0.001	P<0.001	P<0.001	P<0.001

The evaluation of wound infection degree in the two groups of treated patients is described in Tab. IV. (Tab. IV)

**Table 4:** Percentage of infection in ulcers of Group A (CGF) and Group B (controll) at first observation time (T0) and after 3 (T3), 6 (T6) and 9 (T9) weeks.

% of infection	T0	T3	T6	T9
Group A	74%	32%	18%	4%
(n. of pts.)			(28/53)	(3/53)
Group B	65%	58%	42%	34%
(n. of pts.)			(44/47)	(36/47)
p-value for comparisions	p=0.01	p=0.01	p=0.01	p=0.01

No complications or undesirable effects of GCF (such as skin irritation, pain or allergic reactions) have been observed in any of the Group A patients.

### 4.1 Cost-benefit analysis

In the cost / benefit analysis applicable to the use of the CGF for the treatment of difficult ulcers of the lower limbs, the average cost of the CGF application procedure in the study carried out was first evaluated. The costs relating to the application of the CGF consists of the following:

1. Peripheral blood collection (10 cc) in a dedicated sterile test tube: collection was carried out by the nursing staff responsible for dressing the ulcers and only includes the costs of the collection set and the special sterile test tube: 3.15 € (euro).
2. Specific centrifugation with Phase Separator Centrifuge ((Medifuge MF 200® - Silfradent, Italy): the cost of centrifugation is represented by the amortization of the centrifuge and can be considered of about 2.80 € (used for an average of 1500 procedures).
3. Aseptic preparation of CGF for topical application (canvas, gloves and other sterile handling materials): 1.55 €. The total cost of each procedure for applying the CGF as an additional treatment for ulcers of the lower limbs was therefore 7.50 €.

In the analysis, finally, for the time necessary for the procedure with CGF, we must consider on average 3 minutes for the blood collection, 14 minutes for the centrifugation and another 3 minutes for his preparation and application on the wound, for an average total time of 20 minutes. Time spent for the dressing performed with CGF in group A of patients was therefore higher on average by 10 minutes compared to the medication performed in group B of control patients (CGF-free medication, with an average duration of 10 min.). Even if the dressing provided with CGF has a higher unit cost than the standard dressing, the total cost of the treatment is very advantageous since the number of dressings necessary for healing is generally largely reduced.

## 5 Discussion

The presence of a chronic non-healing ulcer is a condition of discomfort for each patient because of the painful symptomatology and the impairments that limit their daily habits in terms of social life or work. The healing of leg ulcers, that are mainly caused by venous insufficiency, makes favourable use of physical methods aimed at the reduction of venous ankle hypertension, whereby the patient extended position, the use of elastic stockings or bandages, combined with the cleaning of the ulcer, leads to healing of the ulcer in variable times [6,17]. The concomitance of obstructive arterial disease of the same limb (also of moderate degree, without critical ischemia) determines a further reduction of the oxygenation of peripheral tissues and determines a greater difficulty and prolongation of healing times, also due to the selective contraindications for elastic compression of the limb [16]. Healing of mixed ulcers of the lower limbs may take several weeks, and several trials indicate the average healing time over 24 weeks, with the frequent risk of early recurrences [20, 21, 22, 23].

The social cost of treatment must also involve the costs of home self- dressing, medical examinations and possible hospitalizations for specific procedures.Furthermore, most patients belong to the category of geriatric patients and may have numerous co-morbidities that influence the course of the wound and interfere with the healing pathway [24].

In fact, the limits of the study can be found in the multi-factoriality of pathologies that support mixed ulcers: they occur especially in the elderly population, both from the point of view of chronic venous insufficiency and for chronic peripheral artery disease, but also for the effect of pharmacological therapies (that each patient practiced at the same time during the study period for these pathologies) or for concomitant chronic pathologies such as diabetes, hypertension and coronary heart disease,: their time of onset, their severity of concomitant diseases, the quantity and quality of associated drug therapies could represent subgroups of study that have not been evaluated in this trial, but have not been considered preventively determinants for the sought therapeutic effect.

A recent controlled critical review, produced with the Cochrane Collaboration method [25], indicates moreover that there is limited evidence to support the fact that dressing modality and their frequency in the management of venous or mixed leg ulcers could be clinically significant on time of healing and on its stability: probably the limited number of trials, the lack of meta-analysis in this field and the poor quality in method does not allow to formulate recommendations with adequate levels of evidence, nor to define the comparative validity of the various types of treatment proposed.

Therapeutic effects of the application of autologous growth factors of blood derivation have found a wide space of application in several sectors of medicine and surgery (from odontostomatology to orthopedics, or plastic surgery and also vascular surgery), both in vitro and in vivo, with various mechanisms: induction of cell proliferation, stimulation of inflammatory cells, increase of collagen deposition and angiogenesis, through the enhancement of enzymatic fibrinolysis and stimulation of protein synthesis [26, 27, 28].

Further analytical and interpretative assessments of the role of autologous growth factors are beyond the scope of this article, but further information can be derived from future studies in this field.The regenerative properties of blood are related to the presence of platelets, leukocytes, mononuclear cells and growth factors. The platelets contain in fact numerous active substances that play an important role in tissue regeneration through the recruitment and activation of mesenchymal cells, such as osteoblasts, fibroblasts and endothelial cells. The active substances are located in different sub-cellular structures: alpha-granules, dense granules, lysosomes and micro-peroxisomes. Alpha-granules contain chemiotactic and mitogenic growth factors important in tissue regeneration, including PDGF, TGF-beta 1 and -beta 2, VEGF, EGF, IGF and FGF. Undoubtedly, personalized compression therapy (especially with low extensibility bandages, possibly through the execution of multi-layer type), in the context of mixed ulcers, and also for those exclusively of venous origin, represents a fundamental step in treatment, and it is reported as a high-grade recommendation in all the guidelines produced in the vascular field.

## 6 Conclusions

The results of this multicentric study on the effects of CGF in the treatment of mixed ulcers of the lower limbs showed a significant advantage in the use of CGF in association with cleansing and selective compression in the healing and stabilization of this kind of ulcers. Pain was also significantly reduced comparatively in the group of patients who were treated with CGF, and furthermore the cost / benefit analysis of the use of this product indicates significant advantages in favour of this procedure. In the treatment of difficult mixed ulcers, CGF has been therefore proved useful and effective in obtaining a faster, less painful, longer lasting and overall cheaper healing of the ulcer. For these reasons, the enhancement of treatment with CGF to all the chronic ulcerative manifestations with a tendency to chronicization is considered valid and suggested by the authors.

## References

[1] Choukroun J., Diss A., Simonpieri A., Girard M.O., Schoeffler C. (2006). Platelet-rich fibrin (PRF): a second-generation platelet concentrate. Part IV: clinical effects on tissue healing. Oral Surg Oral Med Oral Pathol Oral Radiol Endod.

[2] Dohan D.M., Choukroun J., Diss A., Dohan S.L., Dohan A.J. (2006). Plateletrich fibrin (PRF): a second-generation platelet concentrate. Part II: platelet related biologic features. Oral Radiol Endod.

[3] Rodella L.F., Favero G., Boninsegna R., Buffoli B., Labanca M. (2011). Growth factors, CD34 positive cells, and fibrin network analysis in concentrated growth factors fraction. Microsc Res Tech.

[4] Castillo T.N., Pouliot M.A., Kim H.J., Dragoo J.L. (2011). Comparison of growth factor and platelet concentration from commercial platelet-rich plasma separation systems. Am J Sports Med.

[5] Serra R., Gallelli L., Butrico L., Buffone G., Caliò F.G., De Caridi G. (2017). From varices to venous ulceration: the story of chronic venous disease described by metalloproteinases. Int Wound J.

[6] de Franciscis S., Fregola S., Gallo A., Argirò G., Barbetta A., Buffone G. (2016). PredyCLU: a prediction system for chronic leg ulcers based on fuzzy logic; part I - exploring the venous side. Int. Wound J.

[7] Persico G., Amato B., Aprea G., Cerfolio P., Markabaoui A.K. (1995). The early effects of intravenous L-propionyl carnitine on ulcerative trophic lesions of the lower limbs in arteriopathic patients: a controlled randomized study. Drugs Exp Clin Res.

[8] Kobayashi M., Kawase T., Horimizu M., Okuda K., Wolff L.F. (2012). A proposed protocol for the standardized preparation of PRF membranes for clinical use. Biologicals.

[9] Eppley B.L., Woodell J.E., Higgins J. (2012). Platelet quantification and growth factor analysis from platelet-rich plasma: implications for wound healing. Plast Reconstr Surg.

[10] Lucarelli E., Beretta R., Dozza B., Tazzari P.L., O’Connel S.M. (2012). A recently developed bifacial platelet-rich fibrin matrix. Eur Cell Mater.

[11] Weibrich G., Kleis W.K., Hafner G. (2002). Growth factor levels in the platelet-rich plasma produced by 2 different methods: curasan-type PRP kit versus PCCS PRP system. Int J Oral Maxillofac Implants.

[12] Anitua E., Sánchez M., Orive G., Andía I. (2007). The potential impact of the preparation rich in growth factors (PRGF) in different medical fields. Biomaterials.

[13] Passaretti F., Tia M., D’Esposito V., De Pascale M., Del Corso M., Sepulveres R. (2014). Growthpromoting action and growth factor release by different platelet derivatives. Platelets.

[14] Mosesson M.W. (2005). Fibrinogen and fibrin structure and functions. J Thromb Haemost.

[15] Kobayashi M., Kawase T., Horimizu M., Okuda K., Wolff L.F., Yoshie H. (2012). A proposed protocol for the standardized preparation of PRF membranes for clinical use. Biologicals.

[16] Serra R., Amato B., Butrico L., Barbetta A., De Caridi G., Massara M. (2016). Study on the efficacy of surgery of the superficial venous system and of compression therapy at early stages of chronic venous disease for the prevention of chronic venous ulceration. Int Wound J.

[17] Yuan N., Wang C., Wang Y., Yu T., Long Y., Zhang X. (2008). Preparation of autologous platelet-rich gel for diabetic refractory dermal ulcer and growth factors analysis from it.

[18] Okuda K., Kawase T., Momose M., Murata M, Saito Y, Suzuki H (2003). Platelet-rich plasma contains high levels of platelet-derived growth factor and transforming growth factor-beta and modulates the proliferation of periodontally related cells in vitro. J Periodontol.

[19] Yuan N., Wang C., Wang Y., Yu T., Long Y., Zhang X. (2008). Preparation of autologous platelet-rich gel for diabetic refractory dermal ulcer and growth factors analysis from it. Zhongguo Xiu Fu Chong Jian Wai Ke Za Zhi.

[20] Serra R., Grande R., Butrico L., Rossi A., Settimio U.F., Caroleo B. (2015). Chronic wound infections: the role of Pseudomonas aeruginosa and Staphylococcus aureus. Expert Rev Anti Infect Ther.

[21] Amato B., Compagna R., Amato M., Butrico L., Fugetto F., Chibireva M.D. (2016). The role of adult tissue-derived stem cells in chronic leg ulcers: a systematic review focused on tissue regeneration medicine. Int Wound J.

[22] Serra R., Grande R., Buffone G., Molinari V., Perri P., Perri A. (2016). S. Extracellular matrix assessment of infected chronic venous leg ulcers: role of metalloproteinases and inflammatory cytokines. Int Wound J.

[23] Amato B., Coretti G., Compagna R., Amato M., Buffone G, Gigliotti D. (2015). Role of matrix metalloproteinases in non-healing venous ulcers. Int. Wound J.

[24] Serra R., Buffone G., Molinari V., Montemurro R., Perri P, Stillitano D.M. (2015). Low molecular weight heparin improves healing of chronic venous ulcers especially in the elderly. Int. Wound J.

[25] Cooper B., Bachoo P. (2018). Extracorporeal shock wave therapy for the healing and management of venous leg ulcers. Cochrane Database Syst. Rev.

[26] Marx R.E. (2004). Platelet-rich plasma: evidence to support its use. J Oral Maxillofac Surg.

[27] Kobayashi M., Kawase T., Okuda K., Wolff L.F., Yoshie H. (2015). In vitro immunological and biological evaluations of the angiogenic potential of platelet-rich fibrin preparations: a standardized comparison with PRP preparations. Int J Implant Dent.

[28] Anitua E., Sánchez M., Orive G., Andia I. (2008). Delivering growth factors for therapeutics. Trends Pharmacol Sci.

